# The Shadow of Silence on the Sexual Rights of Married Iranian Women

**DOI:** 10.1155/2015/520827

**Published:** 2015-02-01

**Authors:** Roksana Janghorban, Robab Latifnejad Roudsari, Ali Taghipour, Mahmoud Abbasi, Ilsa Lottes

**Affiliations:** ^1^Student Research Committee, Department of Midwifery, School of Nursing and Midwifery, Mashhad University of Medical Sciences, Ebne-Sina Street, Mashhad 9137913199, Iran; ^2^Maternal-Fetal Medicine Research Center, Shiraz University of Medical Sciences, Shiraz 7194634786, Iran; ^3^Research Center for Patient Safety, Department of Midwifery, School of Nursing and Midwifery, Mashhad University of Medical Sciences, Ebne-Sina Street, Mashhad 9137913199, Iran; ^4^Health Sciences Research Center, Department of Biostatistics and Epidemiology, School of Health, Mashhad University of Medical Sciences, Daneshgah Avenue, Mashhad 9137673119, Iran; ^5^Medical Ethics and Law Research Center, Shahid Beheshti University of Medical Sciences, Tehran 1516745811, Iran; ^6^Department of Sociology and Anthropology, University of Maryland, Baltimore County, Baltimore, MD 21250, USA

## Abstract

There has been a recent shift in the field of sexual health, representing a move away from biomedical concerns to sexual rights frameworks. However, few studies on sexuality are based on a rights framework. The unspoken nature of sexuality in Iranian culture has led to a lack of national studies on the topic. The objective of this study was to explore the perceptions and experiences of married Iranian women on sexual rights in their sexual relationships. In this grounded theory study, 37 participants (25 married women, 5 husbands, and 7 midwives) were selected. Data were collected through in-depth interviews and analyzed through open, axial, and selective coding using MAXQDA software version 2007. The analysis revealed the core category of “sexual interaction in the shadow of silence.” The interrelated categories subsumed under the core category included adopting a strategy of silence, trying to negotiate sex, seeking help, and sexual adjustment. The silence originating from women's interactions with their families and society, from girlhood to womanhood, was identified as the core concept in Iranian women's experiences of sexual rights. A focus on husbands' roles seems salient because they can direct or alter some learned feminine roles, especially silence regarding sexual matters, which then affects the realization of women's sexual rights.

## 1. Introduction

Sexuality has been recognized as a central aspect of people's lives and is influenced by biological, cultural, ethical, legal, historical, religious, and spiritual factors [1]. According to the definition given by the World Health Organization (WHO), “sexual health requires a positive and respectful approach to sexuality and sexual relationships, as well as the possibility of having pleasurable and safe sexual experiences, free of coercion, discrimination and violence. For sexual health to be attained and maintained, the sexual rights of all persons must be respected, protected and fulfilled” [2]. Sexual rights were first acknowledged by the global community at the International Conference in Population and Development (ICPD) in Cairo, Egypt, 1994 [3]. These rights were then defined by the Platform for Action of the World Conference on Women in Beijing, 1995, as follows: “The human rights of women include their right to have control over and decide freely and responsibly on matters related to their sexuality” [4]. However, after nearly two decades of formal recognition of sexual rights at international conferences, it has remained subordinate to reproductive rights. Possible reasons for this include the fact that sexual rights are commonly seen as a subset of reproductive rights and considered a more sensitive issue [5, 6]. Efforts are now being made to change this. For example, in 2008 the World Association for Sexual Health (WAS) published its declaration “Sexual Health for the Millennium,” stating that the acceptance of sexual rights was necessary to both promote sexual health and advance the Millennium Development Goals (MDGs). Reproductive and sexual health and rights were at first excluded from the MDGs, but the United Nations (UN) later acknowledged their importance in achieving the goals partly due to pressure from WAS [6].

In recent years the focus on the importance of sexual health and rights has increased markedly, leading the WHO to state that “unsafe sex is the second most important risk factor leading to disability, disease or death in developing countries and the ninth most important in developed countries” [5]. Furthermore, the International Planned Parenthood Federation issued a list of sexual rights as a “Declaration of Sexual Rights” in 2008 [7]. Finally, the importance of sexual rights in the achievement of sexual health is now deemed so important that WAS created this slogan in 2013: “To achieve sexual health, picture yourself owning your sexual rights” [8].

The development of sexual rights requires a greater focus on both its medical and its social aspects. Although the shift from biomedical concerns to sexual rights frameworks has been initiated, there is now a need for studies on sexuality that apply such frameworks [9, 10]. In Iran, the majority of studies on sexual rights have focused on its negative aspects such as problems and diseases; the positive aspects including pleasure and fulfillment have been largely ignored. Additionally, the dominant research approach used in these studies has been based on positivist paradigms [11–17]. A small number of qualitative studies have been conducted on perceptions of sexuality and sexual experiences in Iranian immigrants in the United States, Sweden, Australia, and Canada [18–22]. It seems that the lack of qualitative studies around sexual experiences both inside and outside the country is at least partly due to the unspoken nature of sexuality in Iranian culture. The dominant ideology in Iran is Islam, and the prevalent attitudes toward women's sexual rights are based on long-standing Islamic laws [23]. The sociocultural climate around women's sexuality is defined as “an honorable and valuable feature of femininity which is subject to regulation and protection” [24].

We know that the concept of sexual rights has a broad definition and does not only refer to “the right to have sex” [25]. Thus, the right to consensual sexual relations, the right to pursue satisfying and pleasurable sex, and the right to the expression of sexual desire inside marriage make up the key elements of the sexual rights of individuals [5]. Therefore, this study was conducted to explore the perceptions and experiences of married Iranian women about these aspects of sexual rights in their sexual relationships.

## 2. Methods

A grounded theory approach was used in this study. Grounded theory is an appropriate methodology for exploring little known topics and social processes that affect women's health issues [26, 27]. To our knowledge, the perceptions and experiences of married Iranian women regarding sexual rights are an unexplored subject and not previously highlighted in the literature. A grounded theory approach was adopted in this study because when exploring health-related issues and women's needs, it is important to understand their experiences and to explore the social interactions and situations within which their understanding and insight develop [28].

## 3. Study Setting and Participants

The setting of this study included urban health centers and participants' workplaces in Mashhad, located in Northeast Iran and the country's second largest city. To explore as many aspects and views of married women as possible, the participants were chosen from all five segments of the city's urban health center divisions. According to Iranian health policy, midwives, as counselors, provide sexual counseling for women in these centers.

A total of 37 participants took part in this study: 25 married women whose ages ranged from 19 to 50 years with a mean age of 30.9, five husbands with an age range of 24 to 57 years and a mean age of 39.4, and seven midwives with a range of work experience from 3 to 30 years. Married women who spoke Persian and lived with their husbands were recruited for the study. Women were excluded if they were involved in the divorce process, did not live with their husbands, or did not consent to participate in this study. Maximum variation was considered via the selection of participants of various ages, educational levels, marriage duration, number of children, and occupational status.

## 4. Sampling and Data Collection

Participants' recruitment began with purposive sampling (a method to examine phenomena where it is found to exist [27]). This was followed by theoretical sampling (a process to examine categories and their relationships and to assure that representativeness exists [27]), according to the codes and categories that emerged. First, data collection was conducted via in-depth interviews with married women. After analyzing 10 interviews, the emerged codes and categories were related to the husbands' roles and the quality of sexual health services. Thus, the researchers interviewed husbands and midwives as key informants to improve the theoretical sampling requirements. Once the consent of the participants was obtained, the participants were interviewed face-to-face. Interviews were arranged at a venue most convenient for the participants. To collect data face-to-face, semistructured interviews using an interview guide were conducted. Because of the ambiguity and unfamiliarity of the sexual rights concept, the direct questioning of participants was not possible. Therefore, at the beginning of each interview, participants were asked to explain their own feelings and perceptions about their sexual life. Once participants felt more comfortable talking about this sensitive issue, they were asked related questions such as how they act to express their sexual needs and desires, how they deal with problems in their sexual life, how they became involved in sexual relationships that they did not want, and what conditions affect their behavior in such situations. The interviews continued with probing questions based on the responses of participants. Each interview lasted between 60 and 90 minutes and was recorded and transcribed verbatim.

## 5. Data Analysis

**Figure 1:**
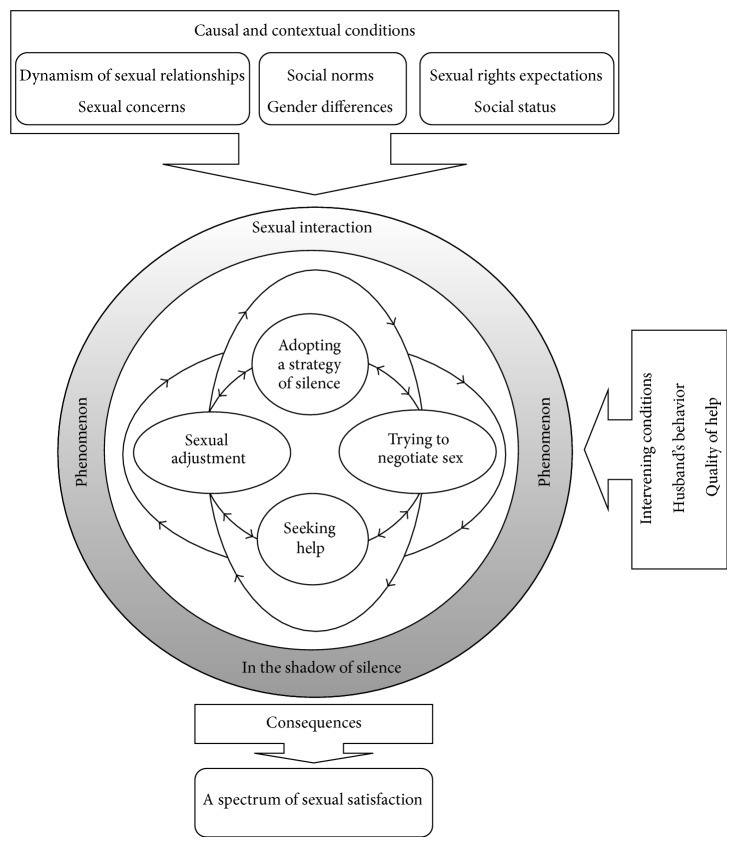
Theoretical scheme of women's perceptions of sexual rights in their sexual relationship.

Data collection and analysis were carried out simultaneously. Data analysis was conducted using Strauss and Corbin's recommended method and involved a constant comparison through three levels of open, axial, and selective coding [27]. In open coding (the process of line-by-line analysis of interview transcripts to reveal and identify concepts and their properties and dimensions in the data [27]), transcripts were read repeatedly and coded line-by-line. The codes were then compared constantly within and between interviews, and then similar codes were grouped together in categories. In this stage, 146 codes, nine subcategories, and four categories were developed. In axial coding (the process of relating categories to their subcategories [27]), categories and subcategories were linked together using a paradigm model, and its different parts, including phenomena, conditions, action/interaction strategies, and consequences, were identified (Figure 1). In selective coding (the process of integrating and refining the theory [27]), the integration of main categories into a unified theoretical explanation and the identification of a core category were conducted using techniques such as writing a story line, making integrative diagrams, and reviewing and sorting memos. In the final stage, the theoretical scheme was outlined and reviewed for internal consistency and gaps in logic. Poorly developed categories were filled and the scheme was validated through its comparison with the raw data. The software MAXQDA 2007 was used for organizing and managing the analysis of data.

## 6. Rigor

The trustworthiness of the research was established according to Lincoln and Guba's key elements of rigor including credibility, confirmability, and transferability [29]. To ensure credibility, various methods including prolonged engagement with participants, writing memos, member checking, and peer debriefing were used. Additionally, participants' recruitment with maximum variation supported the credibility of data. We benefited from data source triangulation with a theoretical sampling of midwives and husbands. Furthermore, the contribution of researchers from different disciplines allowed the exploration of phenomenon with multiple lenses and enhanced the credibility of the analysis.

Throughout the analysis, the consistency of the findings and dependability of the data were promoted by having several researchers who independently coded sets of data and then met to reach consensus on the emerging codes and categories. Two of the authors (Roksana Janghorban and Robab Latifnejad Roudsari) agreed on the way the codes and categories were labeled and categorized; in later stages, these were verified by the other three authors (Ali Taghipour, Mahmoud Abbasi, and Ilsa Lottes). Confirmability was achieved through separate coding by the first and second authors, whereby similarities and differences were discussed. Consensus on codes and subcategories was thereafter achieved. All authors read, discussed, and agreed on the final categorizations.

To ensure transferability, findings were checked and confirmed with a group of women who did not participate in the current study. We tried to document the decision trail of the research in a way that would enable other researchers to follow the research process and establish confirmability.

## 7. Ethical Considerations

The study was approved by the Research Ethics Committee of Mashhad University of Medical Sciences. After a full explanation of the research project, informed written consent was sought from all participants. The confidentiality and anonymity of the participants were preserved. The right to refuse to answer or to withdraw from the study at any time without prejudice was given to all participants.

## 8. Results

This study developed a theoretical scheme for the perceptions of sexual rights held by married Iranian women. The concept of “sexual interaction in the shadow of silence” was the core category and basic social process under which all major categories were subsumed. Four major categories were identified in this process: adopting a strategy of silence, trying to negotiate sex, seeking help, and sexual adjustment.

### 8.1. Core Category

The core category represents the main theme of the research [27]. In this study, the concept of* sexual interaction in the shadow of silence* emerged from participants' experiences and was frequently repeated in the data and related to other major categories. Women described their* sexual interaction* as a process that occurred through several interrelated strategies but placed all under the umbrella of* silence*. In the early stages of marital life, women experienced the dynamism of a sexual relationship and encountered sexual concerns as the causal conditions for the phenomenon of sexual interaction that occurs in the context of gender differences, perceived social norms, sexual rights expectations, and social status. Participants applied a number of strategies for handling the phenomenon. Women often adopted more than one strategy simultaneously. Nearly all women adopted a strategy of silence in terms of expressing their sexual needs and desires at various times in their sexual life, especially in the early stages of marriage. Some then tried to shift their strategy from one of silence to negotiation, seeking help, or sexual adjustment. In this process, some intervening conditions such as a husband's behavior and quality of help received determined whether women were able to continue the adopted strategies or change to another. Women changed these various strategies in their sexual life many times and selected the strategy most appropriate to their situation and even recommended it to other women. Although this process was part of the phenomenon of sexual interaction in the shadow of silence, some strategies had priority over others. None of the strategies followed a chronological pattern; instead they had circular patterns. It is notable that the degree of silence varied from one strategy to another in the process. For instance, the husbands commented that the strategy of silence caused them the greatest concern because the degree of silence adopted by their wives was so high. The silence was evident in strategies, in women's attempts at sexual negotiation through verbal and nonverbal approaches, in their help-seeking behavior from private, professional, and media sources, and in their use of sexual adjustment, which was the consequence of women's sexual sacrificing and pretending to experience sexual satisfaction. The consequence of adopting such action/interaction strategies was the women's perceptions towards their sexual satisfaction. The perception formed from a comparative inference that women made between expected and actual rights in their sexual life (Figure 1). In the following section, the details of the women's purposeful or deliberate acts, which describe the core category, are discussed.

### 8.2. Action/Interaction Strategies

Strategic actions/interactions are tactics that individuals adopt to manage a phenomenon [27]. In this study, the phenomenon was women's sexual interactions with their husbands during their marital life and four interrelated categories considered as action/interaction strategies in the paradigm model: adopting a strategy of silence, trying to negotiate sex, seeking help, and sexual adjustment.

#### 8.2.1. Adopting a Strategy of Silence

One of the first and most common strategies that most women selected to reflect the dynamic nature of their sexual relationship and sexual concerns, as a causal condition, was adopting a strategy of silence.

This strategy was applied through various actions including sexual secrecy and maintaining privacy, as discussed below.

*Sexual Secrecy.* Women stated that they did not talk about their sexual moods, concerns, and feelings with their husbands because of shame, fear of wounding their pride, creating doubt about their chastity, the fear of being labeled as having an excessive sexual drive, and the fear of receiving a negative answer. Their actions originated from their understanding of gender differences in spelling out their needs and the societal norm perceived by women—the internalization of sexual impassiveness. The following extracts confirm these concepts: “*Yeah, sometimes I wanted to have sex, but I did not show that (with emphasis). No, because I always feel that (laugh), you know, I do have pride and I feel that by talking about these issues I would have to swallow my pride*” (Woman 5, 25 years old, length of marriage: 7 years).

Another participant considered that asking for sex might make her husband think that she had premarital sexual relationships, which are frowned upon in traditional Iranian culture. She commented, “*I did not express my desire for sex because I feared that my husband would think that I was too open in our sexual relationship. To be honest, I was fearful that he might think that I'd had previous sexual experience, whereas I had none at all, and believe me, I do not want him to think of me in that way*” (Woman 2, 31 years old, length of marriage: 12 years).

*Maintaining Privacy.* Women maintained their privacy regarding any problems in their sexual relationships, constrained by norms such as the taboo of discussing sex and the belief that sex is a private matter. They set a boundary regarding their privacy so that even the closest family members such as their mothers and sisters were shut out. One woman commented, “*I am very close with my mother and sister, but I do not tell them anything about sex. I never talk about my sexual relationship. I do not allow anybody to ask me about these issues*” (Woman 13, 27 years old, length of marriage: 8 years).

#### 8.2.2. Trying to Negotiate Sex

Over time, some issues led women to change their strategy of silence to one of sexual negotiation, which they recognized as an expected sexual right. Women felt able to be more open in their sexual expression and engage in negotiating by the enhanced intimacy between wife and husband, husbands' behavioral responses such as their attempts to change wives' opinions about their role in sexual relationships, and their interest and willingness to provide the necessary conditions for their wives' expression of sexual desire. A comment by one woman demonstrates this point: “*I can say with confidence that 99 percent of women feel shame when starting a sexual relationship. Meanwhile, my husband convinced me that I could want him. He totally changed my mind towards sexual relationships. However, it took time*” (Woman 3, 28 years old, length of marriage: 5 years).

Regarding their husbands' responsible behavior, women sought to talk with them about sexual issues via verbal disclosure.

*Verbal Sexual Disclosure.* Although women believed in saying the unsaid, the right of mutual enjoyment in a sexual relationship, and the right to express their sexual needs, they found it difficult to have conversations with their husbands about their sexual likes and dislikes or to refuse sex. The main cause of this paradox originates from a key element of Iranian women's femininity, that is, shame and modesty.

*Because I now know that my husband's reaction is positive, I let my husband know when I feel sexual desire. Early in the marriage, I did not tell him because I did not know what my husband's reaction would be. I think that this is my right. I've got the right to be with my husband tonight, to enjoy and to have sex whenever I need to. Well, when I need it, I have to wait till my husband suggests it to me. Why some women ignore it might be because of pride or shame and modesty or an unawareness of their rights and needs. This is both a right and a need that is achievable. (Woman 8, 50 years old, length of marriage: 30 years) *

*Nonverbal Sexual Disclosure.* Shame and the internalization of modesty led to nonverbal communication regarding sexual likes and dislikes or to refuse sex. Nonetheless, the meaning was obvious and understandable by the spouse via behavioral expression and body language.

*I do not say it directly to him, but I think that it detracts from a modest woman's prestige. But I might put on a lot of make-up that night or change my clothes. I wear something that my husband loves. He then sees my desire himself. (Woman 5, 25 years old, length of marriage: 7 years)*

Another participant implied how she refused sex through her nonverbal behaviors: “*Sometimes I do not want to have sex, but you know, I cannot refuse it verbally because of the shame, but somehow I behave in a manner that clearly shows my dislike. Maybe it has previously occurred to you as well that you cannot say something directly to someone, but your behavior is much clearer than saying something*” (Woman 2, 31 years old, length of marriage: 12 years).

The right of sexual negotiation was affected by the husband's behavioral reaction as the main intervening factor. Women's sexual satisfaction was fulfilled following their husbands' sexual responsibility in relation to their wives' desires, needs, and wants. One husband talked about his reaction when his wife did not want sex and refused it: “*I try to consider a shared desire in our sexual relationship. It is very important for me because in my opinion, the enjoyment of sex must be mutual. Therefore, if she refuses sex and I cannot satisfy her, I do not pursue it at that moment*” (Husband 1, 32 years old, length of marriage: 6 years).

However, such negotiations were not always successful or effective. Husbands' unsupportive reactions, including getting angry, not agreeing to or ignoring their wives' requests, and selfishness in sexual relationships, sometimes led to the women experiencing physical and emotional dissatisfaction and the selection of other strategies such as seeking help and sexual adjustment.

#### 8.2.3. Seeking Help

Some women chose to seek help as another strategy in response to the adverse outcomes of sexual negotiation. However, in some cases they did not seek help immediately, and this strategy was adopted following silence or sexual adjustment. Women sought the right to access information and to get help from various sources using this strategy. The various sources of help are presented below.

*Seeking Help from Private Sources.* Seeking help from friends and family members was affected by factors such as the women's level of comfort in talking about sex with others and their families' openness to sexual issues from childhood. These factors influenced who would be the source of help. Typically, most women did not seek help from their mothers but preferred friends or other family members such as a sister. One woman indicated, “*I never speak with my mother about these issues, not even one word [with emphasis]. My mother always maintained privacy and did not let us talk to her or seek her help about sexual issues. She raised us in such a way that we never talk about sex with her*” (Woman 19, 31 years old, length of marriage: 5 years).

Silence due to shame sometimes limited the women's help-seeking process, not only from private sources but also from other sources.

*Well, I bought a book that explained everything. It provided a very detailed explanation about what men and women do in a sexual relationship. I threw it away because of fear of my mom, because I read it during my engagement when I was still living with my mom. Well, I thought that if my mom finds it, she would ask me why are you reading that or why do you want to know that (laugh). I was ashamed in front of her. I still feel ashamed. Then, I threw the book away.* (Woman 5, 25 years old, length of marriage: 7 years).

*Seeking Help from Professional Sources.* A small number of women sought help from experts and professionals such as healthcare providers and sexual counselors. The quality of the help received from professionals affected the success rate of the help-seeking strategy for these women. Often, when visiting health professionals, the women's physical and/or emotional sexual dissatisfaction was not properly addressed because of a lack of professional knowledge, stereotypical approaches, judgment towards clients, refusal to accept the clients' husbands in the process, or only providing help to men: “*I went to the counselor. But she asked me basic stuff; for example, she asked me do you brush your teeth? How she judged me! She wanted to pass the buck to me!*” (Woman 17, 33 years old, length of marriage: 11 years).

Another participant complained that the healthcare providers did not provide gender-sensitive sexual health care: “*I wanted the doctor to make my husband aware of these issues, because I knew it would be better if he heard it from the doctor. I asked the doctor if it would be possible for my husband to come to see her and she said `no, I cannot explain sex to every man'”* (Woman 5, 25 years old, length of marriage: 7 years).

One of the midwives confirmed that healthcare providers are not keen to advise men: “*I do not like to counsel men about sexual problems. Honestly, the main reason that I have chosen midwifery as a profession is that I wanted to deal only with women*” (Midwife 4, length of employment: 20 years).

Additionally, women's shame sometimes made them reluctant to seek help from healthcare providers. One woman said, “*It is very difficult for me to talk about sexual matters with health staff because I feel shame. It's not something that I want to talk about with someone else. I would like to get help from the health center but am I embarrassed*” (Woman 25, 33 years old, length of marriage: 14 years).

Women's general reluctance to receive information and help from health services was confirmed by healthcare providers. Women's behavior led to the adoption of a nonproactive style by health providers to provide help. One midwife said, “*Many women do not even like to talk about sexual issues. You know, when I ask a question in relation to sex, they avoid giving a direct response. They are so embarrassed and some get very upset. They say, that's OK, or do not ask me about these things. Yeah, they have told me this many times. Really, such responses have encouraged me not to ask about sex*” (Midwife 7, length of employment: 18 years).

*Seeking Help from the Media.* The quality of information received from the media had an important role in the fulfillment or violation of women's sexual rights. In some cases, the use of print and digital media and sex education programs for couples based on religious teachings resulted in a greater level of sexual responsibility adopted by husbands and helped the women to achieve satisfaction in their marital life. “*Most men must understand that sometimes, a woman has no desire for sex. Well, at first, my husband did not know such things. “Golbarg” is a TV program where a clergyman speaks very clearly about what you should do in sexual relationships, and how these issues are from the perspective of religion. I saw that he watched it closely. For example, he watched it every Thursday. It was very good and helped us because we understood what we should and should not do, both of us, me and him, as I watched the program with him too*” (Woman 14, 27 years old, length of marriage: 3 years).

However, in a few cases, national and international media had a negative effect on women, and they sought sexual adjustment or returned to silence and help strategies. The damaging effects of media might include a husband's infidelity and exposure to inappropriate sexual patterns (especially through pornographic films), which affected the women's perceptions of their husbands' social status and sexual demands. “*Well, we thought that we might watch movies on satellite TV. In many of these films, different methods are used in sex. You know, it's their job; it's not like having sex in a marital relationship. But my husband asks why is my life different to these films? He compares our marital life with these money-making styles. He gets different ideas about sex from these movies or the internet. Then, he wants me to do these new ways of having sex. He wants to have sex in different ways. So, information from the media made the situation worse, not better*” (Woman 16, 28 years old, length of marriage: 8 years).

#### 8.2.4. Sexual Adjustment

Sexual adjustment was the strategy adopted by women in two situations: failure of the previous strategies or because of the impact of contextual conditions such as social norms, gender differences, religious beliefs, and social status, as perceived by the women. Sexual adjustment occurs through sacrificing sexuality and pretending to be satisfied with their sexual relationships.

*Sexual Sacrificing.* Sexual sacrificing was the most salient adjustment strategy women adopted, not only following the failure of previous strategies, but also in numerous episodes of their sexual life according to sociocultural norms. The women often adopted sexual sacrifice voluntarily or sometimes compulsorily for the following reasons: maintaining their marital life, showing commitment to one's husband (*tamkin*) according to the religious teachings, performing wifely duties, giving priority to her husband's sexual needs over her own, and reducing the possibility of infidelity. The women felt they had no choice but to use the strategy when their husbands' reactions were negative and they demanded sex. These views were confirmed by one wife: “*You know, lastly he is a man. In the current status of our society, meeting a husband's sexual demands is the task of a Muslim woman according to Islamic advice. It should be done in terms of his psychological and emotional needs on the one hand, and the issues that he may confront within society on the other hand. See, social status can affect them very much. Well, when men go out of home, somehow, everything is there for them to have any kind of sexual fun. It is there if they want it, better and worse women than me, you know, who expose themselves to men. Now, women on the street are more stylish than us. Therefore, I think that if his sexual needs and desires are met at home, he does not seek any sort of sexual enjoyment or pleasure outside of the home*” (Woman 18, 40 years old, length of marriage: 14 years).

One of the husbands confirmed that* tamkin* is necessary for a woman if she wants to prevent her husband's infidelity: “*Well, women know that men's main goal of marriage is to have sex and if a woman cannot satisfy her husband, he may seek satisfaction from an affair. Therefore, they should not refuse sex*” (Husband 4, 38 years old, length of marriage: 10 years).

Another participant referred to her sexual sacrificing to make her husband satisfied and content. She said, “*I always try to get along with him because I want to keep him happy. I like that he is satisfied and he does not make a fuss because he is not having sex. I do not like it when he says something, you know, any talk regarding our private sexual issues in front of the kids*” (Woman 7, 34 years old, length of marriage: 9 years).

*Pretending to Be Satisfied.* In a few cases, women showed adjustment behavior by deceiving their husbands and pretending to be satisfied in their sexual relationships. However, the real consequence of adopting such a strategy was sexual dissatisfaction. “*He always asks me, what do you want me to do to make you satisfied? Or he asks have you had an orgasm? I just say yes, whereas I haven't. I pretend to be satisfied because I do not want to upset him, or you know, make him forget me and love somebody else or look down his nose at me or have an affair. I always try to show my pleasure and say, yeah, it was very good. But, generally, I do not enjoy my sexual experiences. It has never been hundred percent enjoyment for me*” (Woman 10, 27 years old, length of marriage: 5 years).

### 8.3. Conditions

A number of conditions (sets of events or occurrences) created the various situations, issues, and problems pertaining to all of the aforementioned women's sexual actions/interactions. Causal conditions led to the emergence of a phenomenon, contextual conditions provided a set of circumstances in which women responded through actions/interactions strategies, and intervening conditions mitigated or altered the impact of causal conditions on the phenomenon [27].

#### 8.3.1. Causal Conditions

The findings of this study illustrate the dynamic nature of sexual relationships, and sexual concerns usually represent sets of events that influence women's sexual rights. Women perceived a sexual dynamism—a temporary increase or decrease in their or their spouse's sexual desires and activities—at certain times in their lives such as in the early years of marriage, pregnancy, and postpartum. Women stated that, despite their feelings, their husbands were eager to have sex in the early stages of marriage. The women did not like to have sex during pregnancy owing to pain and fear of spotting and miscarriage and also during their postpartum period owing to postpartum blues. Additionally, some participants faced sexual concerns including pain during sex, high or low sexual desire, and some sexual dysfunctions in themselves or their husbands. Both perceived sexual dynamism and sexual concerns led to women's diminished pleasure and as a consequence they felt bad about sex and tried to adopt actions/interactions to manage it.

#### 8.3.2. Contextual Conditions

The women's perceptions of sexual rights developed in the context of social norms, gender differences, sexual rights expectations, and social status. They described social norms, such as the taboo of talking about sex and the internalization of female sexual passivity. Most women grew up in circumstances that banned them from talking about sexual issues within their family and induced the belief that men should always enjoy and profit from sexual relationships. On the one hand, women acknowledged gender differences regarding the importance of sex, expressing feelings, insistence for sex when the other spouse was not interested, and providing satisfaction in such situations. On the other hand, they had some expectations as a woman to achieve mutual sexual understanding, enjoyment from desirable relationships, and being satisfied both sexually and spiritually. Additionally, behavioral changes in patterns of friendship with the opposite sex in society induced women to fear their husbands' infidelity. All of these contextual conditions influenced their perceptions of sexual rights and affected their views about sexual interaction.

#### 8.3.3. Intervening Conditions

The quality of help and husbands' behavior as intervening conditions could help explain why some women achieved sexual satisfaction with a selected action/interaction, whereas others did not. For example, husbands' supportive or unsupportive behavior could create a sense of satisfaction or dissatisfaction in women after adopting a strategy of verbal sexual disclosure. The degree of support affected the continuity of the strategy or the selection of another. Women might seek help from professional sources. However, if they received ineffective help and information to resolve problems in their sexual life, they never attempted to seek such help again. It should be noted that the quality of help from the source depended on the care provider's knowledge and other factors in the client-provider interactions.

### 8.4. Consequences

These consequences represent the outcomes of the adopted action/interaction strategies [27]. In this study, women's perceptions regarding the comparison of their expected and actual sexual rights provided a spectrum of consequences from satisfaction to dissatisfaction in physical or/and emotional areas of their sexual life. The expectations of some women were met and they found peace and were satisfied with their sexual life. Their expected sexual rights were fulfilled totally or partially according to the level of their husbands' sexual responsibility. Conversely, other women thought that once married, everything would fall into place. However, they did not have pleasurable sexual experiences and felt a sense of frustration. For a few women, over time, the dissatisfaction led to more undesirable consequences including falling out of love, emotional divorce, and couple burnout.

## 9. Discussion

The major finding of this study is “*sexual interaction in the shadow of silence*.” The women described this concept as a process using various strategies according to their understanding of situations. The actions/interactions consisted of adopting a strategy of silence, trying to negotiate sex, seeking help, and sexual adjustment.

*Adopting a strategy of silence*, which was recognized as the most frequent action/interaction in this research, is deeply rooted in the women's lives. It seems that this strategy originated during childhood when the internalization of sexual passivity was encouraged. In a study by Maasoumi et al. on the perceptions of sexual socialization in Iranian women, women were found to perceive that their parent's conservatism related to children's sexuality issues in the family and that “passive social support systems” existed within society. They found that “passive sexual socialization” was the main societal message related to women's sexuality in Iran [30]. Additionally, Latifnejad Roudsari et al. found that female Iranian adolescents perceived both indirect and direct messages from their parents not to talk about sex [31]. Merghati Khoei similarly introduced silence as a characteristic of the girlhood period before marriage, which can continue to womanhood, even in Iranian immigrant women in Australia [24]. The concept of silence in Asian culture was also confirmed by Gratch et al. A comparison of both Asian college students and non-Asian students showed that the former had a higher level of self-silencing [32]. Additionally, Khoei and Richters indicated that women perceive sexual matters such as sexual relations as private issues between husband and wife and as a private part of their life that should not be discussed in public, even among women who know each other well [33]. We also found that the silence strategy could result in “*maintaining privacy*” in our participants. Bearing in mind this background, the women in our study entered marital life and applied the previously learned strategy to their sexual rights, that is, silence. Thus, they engaged in* sexual secrecy* and* maintaining privacy*.

The silence caused by cultural sexual scripts affected the sexual agency of most of the women in the early years of marriage, and therefore they found it difficult to express their concerns and needs. Thus, they tried to match their sexual relationships with cultural scripts. However, some conditions enabled them to change the interpersonal scripts over time from one of silence to one of negotiation. The behavior of their husbands also acted as a major intervening condition that affected the wives' attitudes towards gender roles and led to a further strategy:* trying sexual negotiation*. This situation helped women to feel better able to assert their own rights and to communicate what they like and dislike during sexual relations. Masters et al. developed three response types to cultural scripts in young boyfriend-girlfriend relationships: conforming, exception-finding, and transforming [34]. Participants in that study were categorized into one of three groups based on behavior that was consistent or different from the sexual scripts. In the present study, a process encompassing continuity and change was seen during marital life. It seems that this shift depended on the quality and depth of intimacy and interactions between the women and their husbands. The findings of this study relate to the roles of the husbands and are consistent with the findings of Wood et al. The findings of the earlier study indicated that postmenopausal women who experienced healthy and respectful relationships with their partners negotiated sexual agency better than those with problematic relationships [35]. In the current study, we detected a trail of silence in the negotiation process such that some women negotiated their sexual agency using a* nonverbal disclosure* strategy because of feelings of shame and the necessity of being a modest woman. A study of Ugandan women by Wolff et al. also reported that the women found it difficult to negotiate sexual matters with their partners and indicated that their negotiation was primarily nonverbal [36]. It must be noted, however, that our study was conducted in a different context in terms of culture, religion, marriage status, and polygamy. Verbal and nonverbal sexual disclosure do not guarantee the fulfillment of women's sexual rights as there were women in the present study who perceived their negotiations to be ineffective and therefore adopted other strategies such as* seeking help* or* sexual adjustment* to protect themselves from having their sexual rights violated by their husband.

*Seeking help* was generally adopted when women could not achieve an effective outcome by talking with their husbands about their sexual concerns. They sought help from various sources including private and professional sources and the media. Indeed, the shame of talking about sexual issues and the fear of judgment hampered the women from getting support from these avenues. Sometimes gender stereotypes related to sex led women to seek help from a family member other than their mothers. Additionally, gender stereotypes limited the women's ability to seek help from media (print media or books). Some studies have reported that the pattern of mother-daughter communication affects the right of adolescents to access information about sexual and reproductive matters [37, 38]. Our study showed that this kind of communication often continues into adulthood and leads to women seeking help from other family members. For the women who sought professional help, the counselor's characteristics played a critical role in whether the women were able to realize their right to access information. A number of factors hindered the ability of counselors to help the women in this study, including counselors' desires to help female clients, using nonproactive styles because of embarrassment, and insufficient knowledge of health professionals about sexual concerns. Thus, these conditions impeded the women's access to information and help. In a recent review on doctor-patient interactions in seeking help for sexual problems in middle and later-life age groups, Hinchliff and Gott reported a preference in both providers and patients to discuss sexual issues with those of the same sex [39]. However, the latter concern by our participants was not a salient issue because most sexual health services are provided by female professionals. Additionally, Hinchliff and Gott found that the doctors' acceptance of some stereotypes such as asexuality in older people prevented them from taking a proactive role. We had no such findings in the current study because of the younger age of our participants.

The women emphasized both positive and negative effects of media on their realization of sexual rights. The effects were strongly related to the content of the programs. With respect to the impact of religious teachings on Iranian culture, women believed that sex education programs from clergies in national media could provide an effective information source for themselves and their husbands. The Global Study of Sexual Attitudes and Behaviors also showed that, among the seven geographical regions of the world, women and men in Middle Eastern countries were most likely to seek help from clergy or religious scholars [40]. Additionally, our participants believed that national mass media reinforces some cultural sexual scripts such as the emphasis on male pleasure instead of both spouses' pleasure. This belief encouraged them to adopt a self-silencing strategy. In relation to this matter, Brown stated that, according to Agenda Setting and Framing Theories, “the media tell people both what is important in the world around them, and how to think about the events and people who inhabit that world” [41]. In addition to the national media, the role of satellite networks in the help-seeking process was salient. Despite the illegality of satellite in Iran, recent reports indicate that between 50% and 70% of Iranian households have access to satellite TV [42]. Few women or their husbands sought help via this source, particularly pornographic films, and women held negative perceptions of the effects of such information on their marital life. Some studies have found pornography to be a threat to marital relationships owing to an increased risk of loss of trust, infidelity, decreased sexual satisfaction with their own partner's love, physical appearance, and sexual performance [43, 44].

The women in this study used the* sexual adjustment* strategy in two ways: as a primary strategy according to sociocultural norms or as a secondary tactic following an unsuccessful previous strategy, that is, seeking help or trying to negotiate sex. The majority of participants primarily adopted* sexual sacrificing*. Most felt some moral obligations to respond to their husbands' sexual needs over their own, which originated from their sociocultural and religious beliefs. In a few cases, the obligation tended towards a compulsion and led to sexual sacrifice as a secondary strategy because those wives feared a repeat of their husband's inappropriate behavior. Wood et al. described how sociocultural assumptions often serve men's sexual needs and affect women's sexual agency [35]. However, our participants' perceptions in an Islamic culture differed from Wood's study; they perceived the wife's sexual submission (*tamkin*) as evidence of performing religious duties and being a devoted Muslim woman, not feeling subordinated. This finding is consistent with Khoei et al. [21]. However, it is noteworthy that most of our participants considered sociocultural norms rather than religious beliefs as the main cause of their submission.

*Pretending to be satisfied* was adopted as a secondary tactic by some of the women. They reached the conclusion, based on previous experience, that expressing internal feelings could sometimes be a threat to their marital relationships. Therefore, they showed outward satisfaction while internally feeling dissatisfaction with their sexual experiences. This concept seems close to a self-silencing schema, that is, a “divided self,” which means that “women feel they have to act in a certain way to please their partner” and therefore enhance “the contrast between their internal and external self” [45].

The main strength of our study is the use of a qualitative approach to elicit women's perceptions and experiences around a sensitive issue, that is, sexual rights, which has only been addressed in a small number of studies in Iran. The study had some limitations. We tried to recruit participants with maximum variations but the findings cannot be generalized to all Iranian women. Although we interviewed men through theoretical sampling, interviewing couples could provide additional insights on sexual rights. It is notable that the majority of women in this study did not agree to be interviewed in the presence of their husbands. They stated that the experiences that have been shared with the researchers in this study have never been disclosed to anybody, and they do not want them disclosed, even to their husbands.

## 10. Conclusions

In this study, the women's experiences in marital life show that they adopted action/interaction strategies to handle sexual rights issues. Thus, they perceived “*sexual interaction in the shadow of silence*” as the core of their experience. The most prominent concept in this process was silence. It seems that this perception reoccurs multiple times in women's interactions with their families and society, from girlhood to womanhood. The role of husbands in marital life was salient because they can direct or alter some of the learned feminine roles, especially those surrounding their wives' silence regarding sexual relationships, and such silence clearly affects the realization of sexual rights. Recognition of this critical issue is essential in the design of a multidimensional program to revise the conservative strategies of family and society in women's sexual socialization, increasing men's participation to encourage their sexual responsibility and assisting healthcare providers and sexual counselors to provide more effective and culture-based sexual healthcare and counseling for couples. Future studies could focus on exploring men's perceptions of sexual rights in connection with the aforementioned program.
